# Supplementary figures and images for: Hair Microbiome Diversity within and across Primate Species
Source: mSystems. 2022 Jul 25;7(4):e00478-22. doi: 10.1128/msystems.00478-22 (PMC9426569; doi:10.1128/msystems.00478-22)

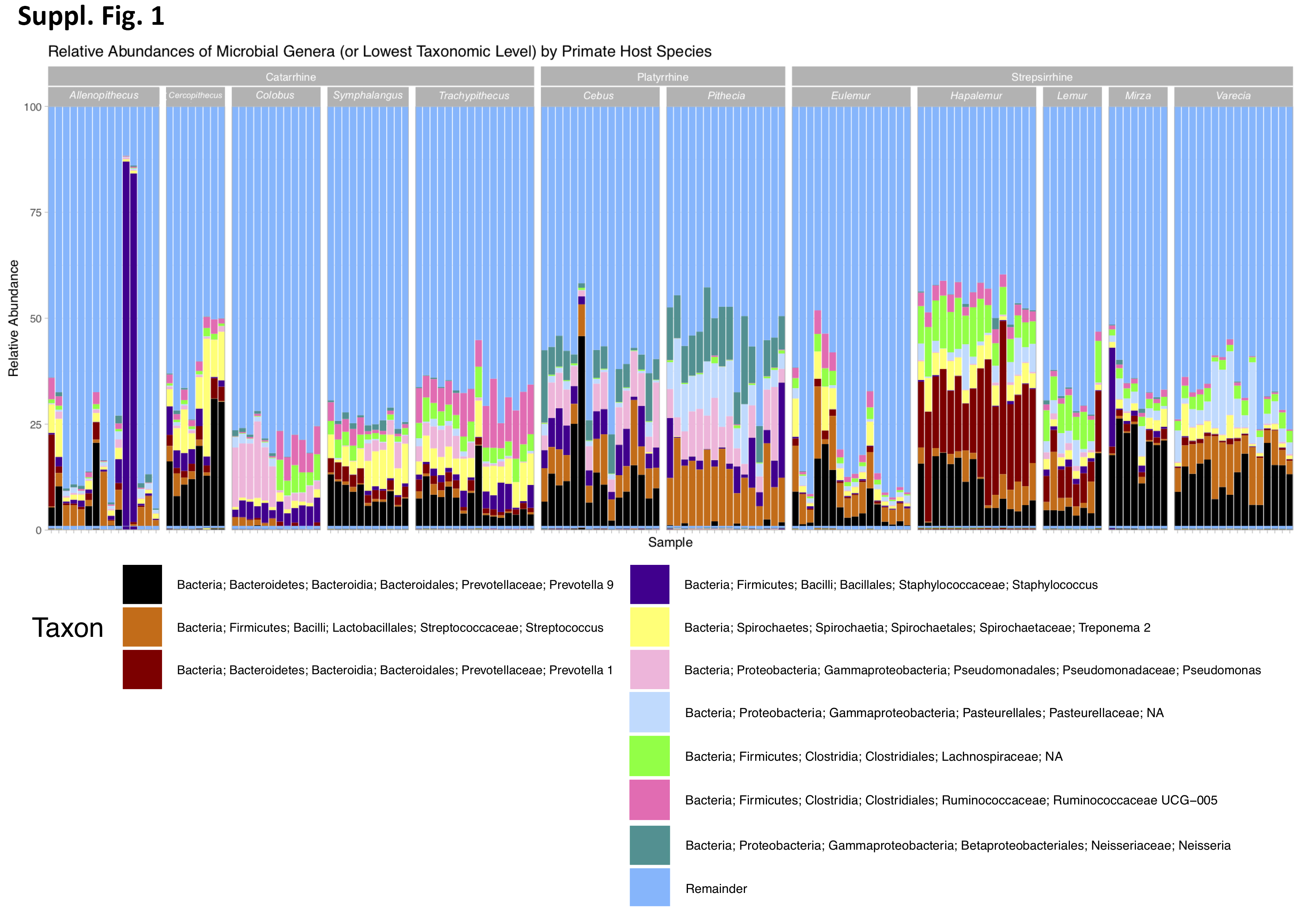

Supplement: FIG S1 [file msystems.00478-22-sf001.jpg]

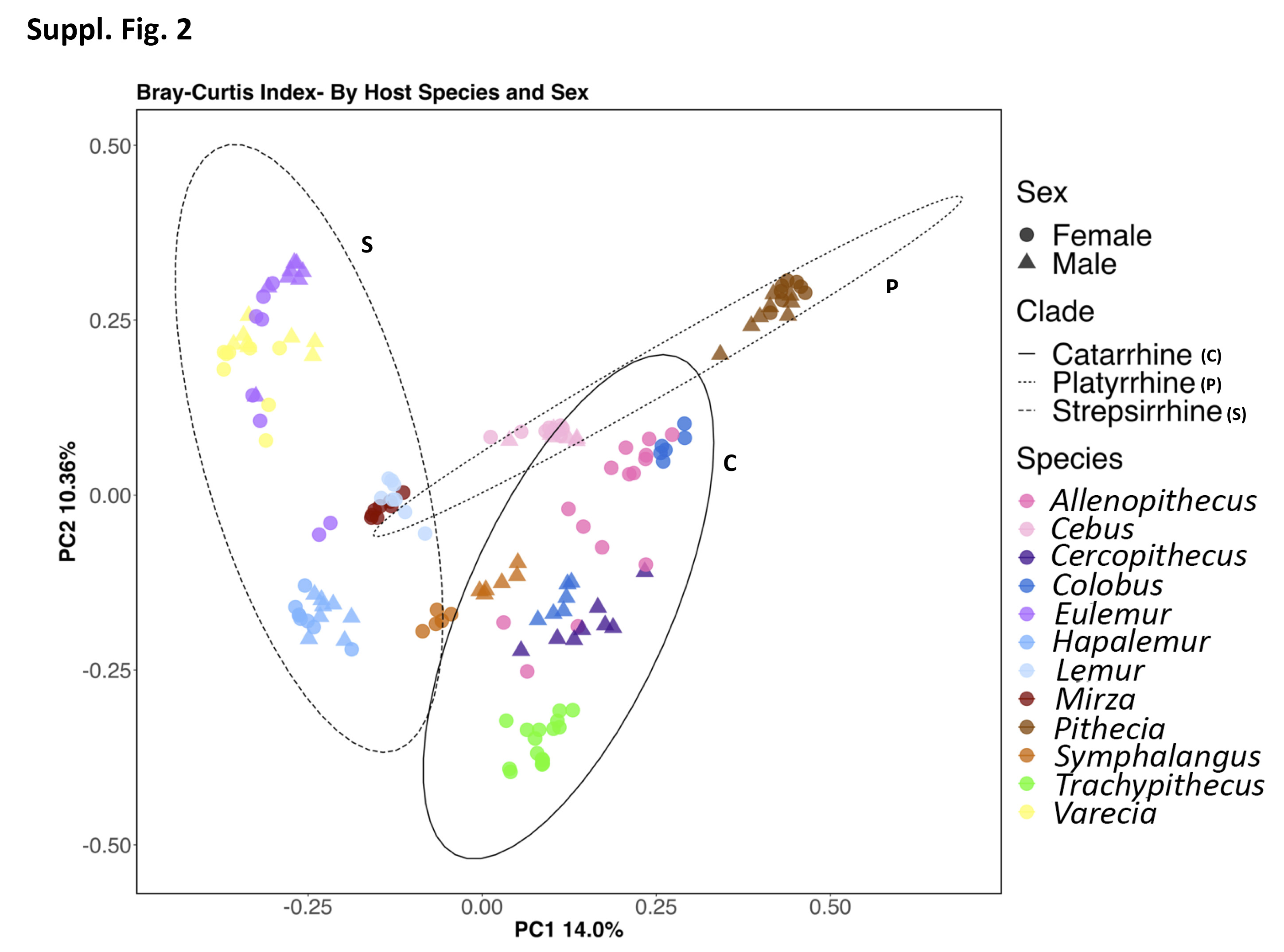

Supplement: FIG S2 [file msystems.00478-22-sf002.jpg]

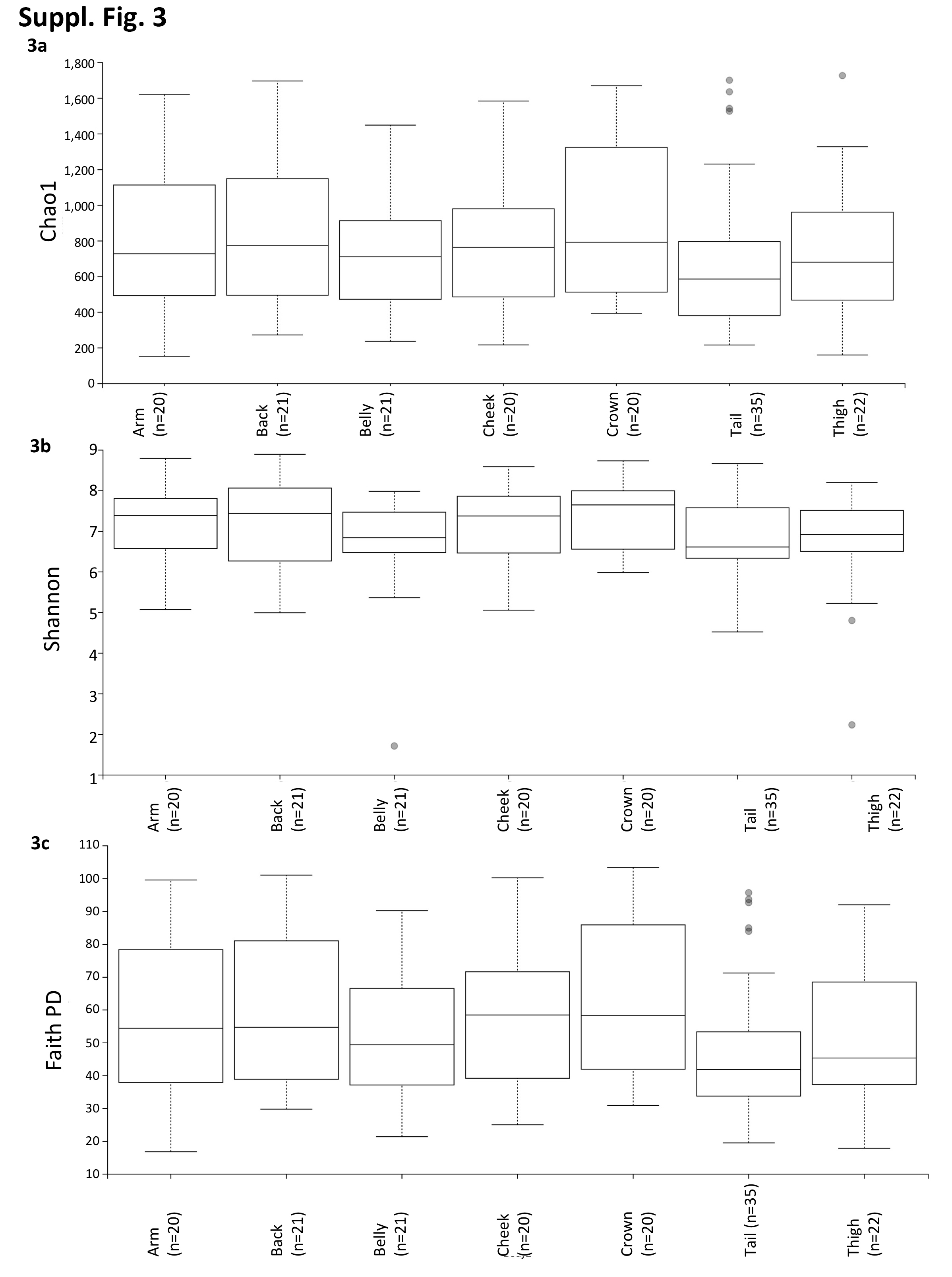

Supplement: FIG S3 [file msystems.00478-22-sf003.jpg]
