# Supplementary material for: Hair Microbiome Diversity within and across Primate Species
Source: mSystems. 2022 Jul 25;7(4):e00478-22. doi: 10.1128/msystems.00478-22 (PMC9426569; doi:10.1128/msystems.00478-22)
Supplement: TABLE S3 [file msystems.00478-22-st003.pdf]

	Allenopithecus	Cebus	Cercopithecus	Colobus	Eulemur	Hapalemur	Lemur	Mirza	Pithecia	Symphalangus	Trachypithecus	Varecia	**Metrics**
Allenopithecus	-------	NS<br>NS<br>.022*<br>NS	.022*<br>.013*<br>.018*<br>NS	.023*<br>.013*<br>.019*<br>NS	.021*<br>NS<br>.021*<br>NS	.020*<br>.011*<br>.018*<br>NS	.002*<br>.004*<br>.005*<br>NS	.005*<br>.012*<br>.004*<br>NS	.023*<br>NS<br>.020*<br>NS	NS<br>.010*<br>NS<br>.003*	.008*<br>.002*<br>.012*<br>.001*	.008*<br>.002*<br>.012*<br>.001*	Chao1<br>Shannon<br>Faith's PD<br>Pielou's
Cebus	-------	-------	.014*<br>.011*<br>.011*<br>NS	.015*<br>.011*<br>.011*<br>NS	.018*<br>NS<br>.020*<br>NS	.013*<br>.009*<br>.009*<br>NS	.001*<br>.004*<br>.001*<br>NS	.005*<br>.012*<br>.002*<br>NS	NS<br>NS<br>NS<br>NS	.026*<br>.008*<br>.014*<br>.004*	.003*<br>.002*<br>.007*<br>.002*	.003*<br>.002*<br>.007*<br>.002*	Chao1<br>Shannon<br>Faith's PD<br>Pielou's
Cercopithecus	-------	-------	-------	NA<br>NA<br>NA<br>NA	.019*<br>NS<br>.023*<br>NS	.001*<br>.004*<br>.001*<br>NS	.011*<br>NS<br>.017*<br>NS	.011*<br>NS<br>.016*<br>NS	.008*<br>.007*<br>.010*<br>NS	NS<br>NS<br>NS<br>NS	NS<br>NS<br>NS<br>NS	NS<br>NS<br>NS<br>NS	Chao1<br>Shannon<br>Faith's PD<br>Pielou's
Colobus	-------	-------	-------	-------	.019*<br>NS<br>.023*<br>NS	.001*<br>.004*<br>.001*<br>NS	.011*<br>NS<br>.017*<br>NS	.007*<br>.014*<br>.002*<br>NS	.008*<br>.007*<br>.010*<br>NS	NS<br>NS<br>NS<br>NS	NS<br>NS<br>NS<br>NS	NA<br>NA<br>NA<br>NA	Chao1<br>Shannon<br>Faith's PD<br>Pielou's
Eulemur	-------	-------	-------	-------	-------	.006*<br>.008*<br>.003*<br>NS	.011*<br>NS<br>.016*<br>NS	.014*<br>NS<br>.008*<br>NS	.012*<br>NS<br>.008*<br>NS	.024*<br>NS<br>NS<br>NS	.025*<br>NS<br>NS<br>NS	.020*<br>NS<br>.023*<br>NS	Chao1<br>Shannon<br>Faith's PD<br>Pielou's
Hapalemur	-------	-------	-------	-------	-------	-------	.011*<br>NS<br>.017*<br>NS	NS<br>NS<br>NS<br>NS	NS<br>NS<br>NS<br>NS	NS<br>NS<br>NS<br>NS	NS<br>NS<br>NS<br>NS	.001*<br>.004*<br>.001*<br>NS	Chao1<br>Shannon<br>Faith's PD<br>Pielou's
Lemur	-------	-------	-------	-------	-------	-------	-------	NS<br>NS<br>NS<br>NS	.001*<br>.003*<br>.001*<br>NS	.004*<br>NS<br>.011*<br>NS	.017*<br>NS<br>.015*<br>NS	.016*<br>NS<br>.014*<br>NS	Chao1<br>Shannon<br>Faith's PD<br>Pielou's
Mirza	-------	-------	-------	-------	-------	-------	-------	-------	.001*<br>.003*<br>.001*<br>NS	.004*<br>NS<br>.011*<br>NS	.017*<br>NS<br>.015*<br>NS	.007*<br>.014*<br>.002*<br>NS	Chao1<br>Shannon<br>Faith's PD<br>Pielou's
Pithecia	-------	-------	-------	-------	-------	-------	-------	-------	-------	.017*<br>.006*<br>.013*<br>.005*	.002*<br>.001*<br>.006*<br>.002*	.005*<br>.012*<br>.005*<br>NS	Chao1<br>Shannon<br>Faith's PD<br>Pielou's
Symphalangus	-------	-------	-------	-------	-------	-------	-------	-------	-------	-------	.010*<br>NS<br>NS<br>NS	.009*<br>NS<br>.023*<br>NS	Chao1<br>Shannon<br>Faith's PD<br>Pielou's
Trachypithecus	-------	-------	-------	-------	-------	-------	-------	-------	-------	-------	-------	.007*<br>.014*<br>.017*<br>NS	Chao1<br>Shannon<br>Faith's PD<br>Pielou's
Varecia	-------	-------	-------	-------	-------	-------	-------	-------	-------	-------	-------	-------	Chao1<br>Shannon<br>Faith's PD<br>Pielou's
