# Supplementary material for: Hair Microbiome Diversity within and across Primate Species
Source: mSystems. 2022 Jul 25;7(4):e00478-22. doi: 10.1128/msystems.00478-22 (PMC9426569; doi:10.1128/msystems.00478-22)
Supplement: TABLE S4 [file msystems.00478-22-st004.pdf]

	Allenopithecus	Cebus	Cercopithecus	Colobus	Eulemur	Hapalemur	Lemur	Mirza	Pithecia	Symphalangus	Trachypithecus	Varecia
Allenopithecus	-------	.011*<br>NS<br>NS	.014*<br>.016*<br>NS	.014*<br>.016*<br>NS	.017*<br>.014*<br>NS	.014*<br>.014*<br>NS	NS<br>.017*<br>.008*	NS<br>NS<br>.007*	.002*<br>NS<br>.011*	.009*<br>.007*<br>NS	.017*<br>.002*<br>NS	.018*<br>.002*<br>NS
Cebus	.017*<br>.001*<br>.001*	-------	.005*<br>NS<br>NS	.005*<br>NS<br>NS	.016*<br>NS<br>NS	.005*<br>NS<br>NS	.012*<br>NS<br>NS	.011*<br>NS<br>NS	.008*<br>.012*<br>.003*	.001*<br>.015*<br>NS	.004*<br>.003*<br>.011*	.004*<br>.003*<br>.011*
Cercopithecus	NS<br>NS<br>.008*	.010*<br>.001*<br>.001*	-------	NA<br>NA<br>NA	.020*<br>.005*<br>NS	.003*<br>.013*<br>.002*	NS<br>NS<br>NS	.020*<br>NS<br>.013*	.001*<br>.08*<br>.010*	NS<br>NS<br>NS	NS<br>.009*<br>NS	NS<br>.009*<br>NS
Colobus	NS<br>NS<br>.008*	.010*<br>.001*<br>.001*	NA<br>NA<br>NA	-------	.020*<br>.005*<br>NS	.003*<br>.013*<br>.002*	NS<br>NS<br>NS	.010*<br>NS<br>.006*	.001*<br>.008*<br>.010*	NS<br>NS<br>NS	NS<br>.009*<br>NS	NA<br>NA<br>NA
Eulemur	NS<br>NS<br>NS	NS<br>NS<br>NS	NS<br>NS<br>NS	NS<br>NS<br>NS	-------	.001*<br>.002*<br>.001*	.020*<br>NS<br>.012*	.007*<br>.005*<br>.004*	.006*<br>.006*<br>.009*	NS<br>NS<br>NS	NS<br>.008*<br>NS	.020*<br>.005*<br>NS
Hapalemur	.017*<br>NS<br>.010*	.008*<br>.001*<br>.009*	.001*<br>.001*<br>.011*	.001*<br>.001*<br>.011*	.001*<br>.001*<br>NS	-------	NS<br>NS<br>NS	NS<br>NS<br>NS	NS<br>NS<br>NS	NS<br>NS<br>NS	NS<br>NS<br>NS	.003*<br>.013*<br>.002*
Lemur	.003*<br>NS<br>NS	.001*<br>.001*<br>.002*	.011*<br>NS<br>.014*	.011*<br>NS<br>.014*	.009*<br>NS<br>.014*	.012*<br>NS<br>.014*	-------	NS<br>NS<br>NS	.002*<br>.011*<br>.002*	.013*<br>NS<br>NS	NS<br>.005*<br>.005*	NS<br>.011*<br>.005*
Mirza	.002*<br>NS<br>NS	.001*<br>.005*<br>.011*	.009*<br>NS<br>.013*	.001*<br>.006*<br>.012*	.001*<br>.008*<br>NS	NS<br>.011*<br>NS	NS<br>.011*<br>NS	-------	.002*<br>.011*<br>.002*	.013*<br>NS<br>NS	NS<br>.005*<br>.005*	.010*<br>NS<br>.006*
Pithecia	NS<br>.004*<br>.004*	NS<br>.010*<br>NS	.014*<br>.002*<br>.001*	.014*<br>.002*<br>.001*	.013*<br>.007*<br>NS	NS<br>.008*<br>NS	.002*<br>.002*<br>.005*	.002*<br>.002*<br>.005*	-------	.001*<br>.004*<br>.008*	.001*<br>.001*<br>NS	.008*<br>NS<br>NS
Symphalangus	NS<br>.003*<br>NS	.011*<br>.001*<br>.002*	NS<br>.012*<br>.008*	NS<br>.012*<br>.008*	NS<br>.009*<br>NS	NS<br>.011*<br>NS	.005*<br>.013*<br>NS	.005*<br>.013*<br>NS	.014*<br>.001*<br>.005*	-------	.019*<br>.010*<br>NS	.015*<br>NS<br>NS
Trachypithecus	.016*<br>.005*<br>.003*	.004*<br>.001*<br>.001*	NS<br>.016*<br>NS	NS<br>.017*<br>NS	NS<br>.014*<br>NS	NS<br>.014*<br>NS	.006*<br>.017*<br>.007*	.006*<br>.017*<br>.007*	.008*<br>.001*<br>.001*	NS<br>NS<br>.006*	-------	.010*<br>NS<br>.006*
Varecia	.015*<br>.005*<br>.003*	.004*<br>.001*<br>.001*	NS<br>.017*<br>NS	NA<br>NA<br>NA	NS<br>NS<br>NS	.001*<br>.001*<br>.011*	.005*<br>.015*<br>.009*	.001*<br>.006*<br>.012*	.007*<br>.005*<br>.011*	NS<br>NS<br>.011*	.001*<br>.006*<br>.012*	-------
