# Supplementary material for: Hair Microbiome Diversity within and across Primate Species
Source: mSystems. 2022 Jul 25;7(4):e00478-22. doi: 10.1128/msystems.00478-22 (PMC9426569; doi:10.1128/msystems.00478-22)
Supplement: TABLE S5 [file msystems.00478-22-st005.pdf]

UniFrac	Independent Variable	P	P_{adj}
Weighted	Species	0.001	---
	Sex	0.010	---
	Institution	0.001	0.0015 (Duke-FW)<br>0.0015 (Duke-GPZ)<br>0.0020 (FW-GPZ)
	Body	0.557	---
Unweighted	Species	0.001	---
	Sex	0.001	---
	Institution	0.001	0.001 (all pairwise)
	Body	0.995	---
