# Supplementary material for: Hair Microbiome Diversity within and across Primate Species
Source: mSystems. 2022 Jul 25;7(4):e00478-22. doi: 10.1128/msystems.00478-22 (PMC9426569; doi:10.1128/msystems.00478-22)
Supplement: TABLE S6 [file msystems.00478-22-st006.pdf]

	Back	Belly	Cheek	Crown	Tail	Thigh	Metric
Arm	*NS*	*NS*	*NS*	*NS*	*NS*	*NS*	*Chao1 Diversity*
	NS	*NS*	*NS*	*NS*	*NS*	*NS*	*Shannon Index*
	NS	*NS*	*NS*	*NS*	**0.007***	*NS*	*Faith's PD*
	NS	**0.002***	*NS*	*NS*	*NS*	*NS*	*Pielou's Index*
Back		*NS*	*NS*	*NS*	**0.005***	*NS*	*Chao1 Diversity*
		NS	*NS*	*NS*	*NS*	*NS*	*Shannon Index*
	-----	*NS*	*NS*	*NS*	**0.005***	**0.017***	*Faith's PD*
		NS	*NS*	*NS*	*NS*	*NS*	*Pielou's Index*
Belly			*NS*	*NS*	*NS*	*NS*	*Chao1 Diversity*
	-----		*NS*	*NS*	*NS*	*NS*	*Shannon Index*
		-----	*NS*	**0.014***	*NS*	*NS*	*Faith's PD*
			NS	**0.005***	*NS*	*NS*	*Pielou's Index*
Cheek				*NS*	*NS*	*NS*	*Chao1 Diversity*
	-----	-----		*NS*	*NS*	*NS*	*Shannon Index*
			-----	*NS*	**0.012***	*NS*	*Faith's PD*
				NS	*NS*	*NS*	*Pielou's Index*
Crown					**0.002***	*NS*	*Chao1 Diversity*
	-----	-----	-----		*NS*	*NS*	*Shannon Index*
				-----	**0.010***	**0.004***	*Faith's PD*
					NS	*NS*	*Pielou's Index*
Tail						*NS*	*Chao1 Diversity*
	-----	-----	-----	-----		*NS*	*Shannon Index*
					-----	*NS*	*Faith's PD*
						NS	*Pielou's Index*
