# Supplementary material for: Hair Microbiome Diversity within and across Primate Species
Source: mSystems. 2022 Jul 25;7(4):e00478-22. doi: 10.1128/msystems.00478-22 (PMC9426569; doi:10.1128/msystems.00478-22)
Supplement: TABLE S7 [file msystems.00478-22-st007.pdf]

	Arm	*Back*	*Belly*	*Cheek*	*Crown*	*Tail*	*Thigh*
Arm	 -----	*NS*<br>*NS*	*NS*<br>*NS*	*NS*<br>*NS*	*NS*<br>*NS*	***0.005****<br>*NS*	*NS*<br>*NS*
Back	*NS*<br>*NS*	 -----	*NS*<br>*NS*	*NS*<br>*NS*	*NS*<br>*NS*	***0.002****<br>*NS*	*NS*<br>*NS*
Belly	*NS*<br>*NS*	*NS*<br>*NS*	 -----	*NS*<br>*NS*	*NS*<br>*NS*	***0.010****<br>*NS*	*NS*<br>*NS*
Cheek	*NS*<br>*NS*	*NS*<br>*NS*	*NS*<br>*NS*	 -----	*NS*<br>*NS*	***0.007****<br>*NS*	*NS*<br>*NS*
Crown	*NS*<br>*NS*	*NS*<br>*NS*	*NS*<br>*NS*	*NS*<br>*NS*	 -----	***0.014****<br>*NS*	*NS*<br>*NS*
Tail	***0.007****<br>***0.002****	***0.005****<br>***0.005****	*NS*<br>***0.012****	***0.010****<br>***0.010****	***0.002****<br>***0.007****	 -----	***0.012****<br>*NS*
Thigh	*NS*<br>*NS*	*NS*<br>*NS*	*NS*<br>*NS*	*NS*<br>*NS*	***0.012****<br>*NS*	*NS*<br>***0.014****	 -----
