# Supplementary material for: Hair Microbiome Diversity within and across Primate Species
Source: mSystems. 2022 Jul 25;7(4):e00478-22. doi: 10.1128/msystems.00478-22 (PMC9426569; doi:10.1128/msystems.00478-22)
Supplement: TABLE S1 [file msystems.00478-22-st001.pdf]

Name/ID #	Species	Common Name	Sex	Institution	Body Site								Clade	Year Sample Collected	Month Sample Collected
					Arm	Back	Belly	Cheek	Crown	Thigh	Distal Tail	Proximal Tail			
97627	*Allenopithecus nigroviridis*	Allen's Swamp Monkey	F	Ft. Wayne Children's Zoo	X	X	X	X	X	X	X	X	Catarrhine	2011	Jan
97737	*Allenopithecus nigroviridis*	Allen's Swamp Monkey	F	Ft. Wayne Children's Zoo	X	X	X	X	E**	X	X	X	Catarrhine	2010	Dec
9331	*Colobus angolensis*	Angola colobus	F	Gladys Porter Zoo	X	X	X	X	X	XX*			Catarrhine	2010	Jul
6041	*Colobus angolensis*	Angola colobus	M	Gladys Porter Zoo		X	X		X	X	X		Catarrhine	2010	Jul
97746	*Cercopithecus neglectus*	De Brazza's monkey	M	Ft. Wayne Childrens Zoo	X	X	X	X	X	X	X	X	Catarrhine	2012	Oct
98357	*Symphalangus syndactylus*	Siamang	F	Ft. Wayne Childrens Zoo	X	X	X	X		X			Catarrhine	2010	Sep
97525	*Symphalangus syndactylus*	Siamang	M	Ft. Wayne Childrens Zoo	X	X	X	X	X	X			Catarrhine	2011	Oct
98114	*Trachypithecus obscurus*	Dusky leaf monkey	F	Ft. Wayne Childrens Zoo	X	X	X	X	X	X	X	X	Catarrhine	2011	Mar
97346	*Trachypithecus obscurus*	Dusky leaf monkey	F	Ft. Wayne Childrens Zoo	X	X	X	X	X	X	X	X	Catarrhine	2011	Jan
97075	*Cebus capucinus*	White-faced capuchin	M	Ft. Wayne Childrens Zoo	X	X	X	X	X	X	X	X	Platyrrhine	2011	Mar
90017	*Cebus capucinus*	White-faced capuchin	F	Ft. Wayne Childrens Zoo	X	X	X	X	X	X	X	X	Platyrrhine	2011	Mar
97063	*Pithecia pithecia*	White-faced Saki	M	Ft. Wayne Childrens Zoo	X	X	X	X	X	X	X	X	Platyrrhine	2011	Mar
97037	*Pithecia pithecia*	White-faced Saki	F	Ft. Wayne Childrens Zoo	X	X	X	X	X	X	X	X	Platyrrhine	2011	Jan
1369	*Hapalemur griseus*	Eastern Lesser Bamboo Lemur	F	Duke Lemur Center	X	X	X	X	X	X	X	X	Strepsirrhine	2006	May
1333	*Hapalemur griseus*	Eastern Lesser Bamboo Lemur	M	Duke Lemur Center	X	X	X	X	X	X	X	X	Strepsirrhine	2006	Unknown
98271	*Lemur catta*	Ring-tailed lemur	F	Ft. Wayne Childrens Zoo	X	X	X	X	X	X	X	X	Strepsirrhine	2011	Oct
6521	*Eulemur flavifrons*	Blue eyed black lemur	F	Duke Lemur Center	X	X	X	X	X	X	X	X	Strepsirrhine	2006	Feb
6407	*Eulemur flavifrons*	Blue eyed black lemur	M	Duke Lemur Center	X	X	X	X	X	X	X	X	Strepsirrhine	2006	Feb
6206	*Varecia rubra*	Red ruffed Lemur	M	Duke Lemur Center	X	X	X	X	X	X	X	X	Strepsirrhine	2006	Feb
6205	*Varecia rubra*	Red ruffed Lemur	F	Duke Lemur Center	X	X	X	X	X	X	X	X	Strepsirrhine	2006	Feb
2322	*Mirza coquereli*	Coquerel's Giant Mouse Lemur	F	Duke Lemur Center	X	X	X	X	X	X	X	X	Strepsirrhine	2006	Jun

*Two thigh samples were taken from this individual
** This sample was excluded in downstream analyses
